# Impacted Canine in Orthodontic Patients of a Tertiary Care Hospital: A Descriptive Cross-sectional Study

**DOI:** 10.31729/jnma.6545

**Published:** 2021-12-31

**Authors:** Bashu Dev Pant, Anjana Rajbhandari, Resina Pradhan, Manju Bajracharya, Pushkar Manandhar, Surendra Maharjan, Dilli Bahadur Pun, Barun Kumar Sah

**Affiliations:** 1 Department of Orthodontics, People's Dental College and Hospital, Kathmandu, Nepal; 2 Department of Physiology, Karnali Academy of Health Sciences, Jumla, Nepal; 3 Department of Pediatric and preventive Dentistry, Institute of Medicine, Kathmandu, Nepal

**Keywords:** *impacted*, *mandible*, *maxilla*, *prevalence*, *tooth*

## Abstract

**Introduction:**

Impacted canine is a frequently encountered clinical problem during orthodontic practice with different prevalence in each region. Treatment usually requires an interdisciplinary approach which is difficult and time consuming. Surgical exposure of the impacted tooth and the complex orthodontic mechanisms are used to align the tooth into the arch. This study was designed to find out the prevalence of impacted canine in orthodontic patients.

**Methods:**

A descriptive cross-sectional study was done with hospital records of patients from 15 to 38 years of age visited department of Orthodontics at a tertiary care hospital from August 2020 to March 2021 after obtaining ethical clearance from the institutional review committee. Convenience sampling method was used. Patients Orthopantomogram and clinical notes were thoroughly evaluated. To locate position of impacted canine cone-beam computed tomography images were used. The data was collected and entered in Microsoft Excel. Point estimate at 99% Confidence Interval was calculated along with frequency and proportion for binary data.

**Results:**

Out of 1008 patients, 44 (4.37%) (2.71-6.03 at 99% Confidence Interval) patients had impacted canines. Among them, maxillary canine impaction was seen in 38 (3.77%) and mandibular canine impaction in 6 (0.60%).

**Conclusions:**

The overall prevalence of impacted canines was found lower than previous studies done in similar settings. Large number of patients had buccal impaction compared to palatal impaction.

## INTRODUCTION

Canines are considered as the cornerstones of the mouth and main roles in the aesthetics and function. The maxillary canines are the most commonly impacted teeth after third molars.^1–3^ The incidence of impacted maxillary canine is said to be 0.9-2.2% while as mandibular canine impaction is two to twenty times lesser.^4-7^ Palatal impaction occurs in 85% of cases frequently in females.^8,9^ Unilateral canine impaction is most common.^10,11^

Impacted or displaced canine is a common problem encountered during orthodontic treatment and it usually involves surgical exposure followed by orthodontic traction to bring the tooth into the occlusion.

The objective of the present study was to determine the prevalence of impacted canine in orthodontic patients of a tertiary care hospital.

## METHODS

This descriptive cross-sectional study was conducted among orthodontic patients in the department of Orthodontics, Peoples Dental College and Hospital, Kathmandu, Nepal, from August 2020 to March 2021 using patient records. Written consent was taken from each participant before pre-treatment record collection. Ethical approval for the study was obtained from the Institutional Review Committee of Institute of Medicine (Ref. no. 22(6-11 )E^2^/077/078). The inclusion criteria of the study were age at least 15 years, presence of impacted permanent canine and no history of previous orthodontic treatment. The exclusion criteria were extraction of maxillary canine, systemic disease, developmental anomalies or syndromes, trauma or fracture of the jaw that might have affected normal growth of the dentition and distorted or poor quality Orthopantomograms with low visibility in canine region. Convenience sampling method was used. Sample size was calculated using the formula,

n = Z^2^ × p × q / e^2^

  = (2.58)^2^ × (0.056) × (1-0.056) / (0.02)^2^

  = 875

Where,

n= required sample size
Z= 2.58 at 99% Confidence Interval (CI)
p= past prevalence of impacted canine taken from a previous study, 5.6%
e= margin of error, 2%

However, 1008 patients were enrolled in the study.

Teeth were considered impacted when they were not completely erupted to the normal functional level in the occlusal line. Orthopentomogram (Sirona Orthophos SL. exposed at 73KV-15mA to 84KV-13mA) and clinical notes were used for identification of impaction. Those patients with a Orthopantomogram and clinical notes suggesting impacted canines were further investigated with a cone beam CT scan machine to locate the position.

The data was collected, tabulated and statistically analyzed descriptively using Microsoft Excel to find out the prevalence of impacted canine in the orthodontic population. Point estimate at 99% Confidence Interval was calculated along with frequency and proportion for binary data.

## RESULTS

Out of the medical records of 1008 patients examined, impacted canines were seen in 44 (4.37%) (2.71-6.03 at 99% Confidence IntervaI) patients. Among them, 406 (40.28%) were male and 602 (59.72%) were females (Table 1).

**Table 1:** Distribution of canine impaction.

Gender	Number of patients examined n (%)	Number of patients with impacted canine n (%)
Male	406 (40.28)	18 (40.91)
Female	602 (59.72)	26 (59.09)
Total	1008 (100)	44 (100)

Out of these 44 patients with impacted canines, 38 (3.77%) were observed in maxilla and 6 (0.60%) were in the mandible (Table 2).

**Table 2:** Distribution of patients with impacted canine according to arch.

Arch	Number of patients with impacted canine n (%)
Maxilla	38 (3.77)
Mandible	6 (0.60)
Total	44 (4.37)

In the maxilla, more number of impactions were noted on left side 17 (38.64%) compared to the right side 13 (29.55%) but on the mandible it was reverse i.e. more patients had impacted canines on right side 3 (6.82%) compared to left side 2 (4.55%) and nine patients had bilaterally impacted canines (Table 3).

**Table 3:** Distribution of patients with impacted canines according to side.

Gender	Maxilla			Mandible			Total n (%)
	Right n (%)	Left n (%)	Bilateral n (%)	Right n (%)	Left n (%)	Bilateral n (%)	
Male	3 (16.67)	10 (55.56)	1 (5.56)	2 (11.11)	2 (11.11)	0	18 (40.91)
Female	10 (38.46)	7 (26.92)	7 (26.92)	1 (3.85)	0	1 (3.85)	26 (59.09)
Total	13 (29.55)	17 (38.64)	8 (18.18)	3 (6.82)	2 (4.55)	1 (2.27)	44 (100)

More number of canine impactions was observed on buccal aspect of both the arch 26 (59.09%) compared to palatal aspect 18 (40.91%). In the mandible female patients had equal number (one) of impactions (Table 4).

**Table 4:** Distribution of patients with impacted canines according to position.

Gender	Maxilla		Mandible		Total n (%)
	Buccal n (%)	Palatal n (%)	Buccal n (%)	Palatal n (%)	
Male	6 (33.33)	8 (44.44)	3 (16.66)	1 (5.56)	18 (40.91)
Female	16 (61.53)	8 (30.76)	1(3.84)	1 (3.84)	26 (59.09)
Total	22 (50)	16 (36.36)	4(9.09)	2 (4.55)	44 (100)

## DISCUSSION

The canine tooth has long and torturous path of eruption from the floor of the orbit to the functional occlusion so, it has most complicated eruption pattern and is one of the last teeth to erupt in dental arch. According to these conditions, this tooth may not have an eruption process in a natural way. The knowledge of dental anomalies is important for prevention, diagnosis, treatment planning, and follow-up. In an orthodontic practice impacted canines constitute a clinical challenge depending upon its severity of impaction so, precise location of these impacted canines is important to formulate the best treatment plan for its eruption into occlusion.

In the present study the prevalence of impacted permanent canine was found to be 4.37% which was lower than the study done in Nepalese population by Piya, et al.^12^ Who reported a prevalence of 5.6% but more than the study done in central Indian population by Jain, et al.^6^ Who reported 1.38%; it may be due to difference in the methodology of study, sample size, radiographic and clinical interpretation and grouping method.

Canine impaction is more frequent in the maxilla than the mandible. The prevalence of maxillary canine impaction in our study was 3.77%, which was in harmony with a study of Riyadh, Saudi Arabia (3.65%) and Turkey (3.58%).^13,14^ But it is lower than the study done in Nepalese population (5.29%) by Upadhyaya and Kafle.^15^ A lower prevalence was recorded in Swedish population (1.7%) in a study done by Ericson and Kurol^16^ 2.94% prevalence in Turkey^2^ and 1.62% among Kosovar patients.^17^ Higher incidences have been reported with 8.8% in Greece and 6.04% in Mexican population.^3,4^

The prevalence of impacted mandibular canine in this study was found to be 0.6%, which was higher than the study done by Jain, et al. (0.37%) and Grover, et al. (0.22%).^6,18^ Most of the studies published on literature have dealt with characteristics of unilateral impaction.^10,19^ Our results also support this conclusion; in our study out of 44 patients 35 patients have unilateral impaction (79.55%). Most commonly impacted side was left one.^20,21^ which is similar to present study; in which 19 patients (43.18%) have left side impaction, 16 patients (36.36%) have right side impaction and nine patients (20.45%) have bilateral impaction.

When it comes to the prevalence of impacted canine according to the gender then most of the studies reported higher prevalence among the females.^5,22^ Equal occurrence of impacted canine in male and female was reported by few studies.^23,24^ In the present study we have also found almost equal prevalence among both the genders.

In a European population, palatal canine impaction was around five times more frequent than the Asian population and it was genetic in origin.^25^ In our study overall 26 (59.09%) patients have buccal impaction and 18 (40.91%) patients have palatal impaction. These differences may be due to racial differences in jaw bone structure between European and Asian population.

Gender-wise data showed that the maxilla male patients have more palatal impaction (eight) compared to female patients who have more buccal impaction (16). It may be due to smaller cranium in the female patients compared to male, which may lead to decrease in the facial skeleton and jaw size due to which there is arch length deficiency and causes buccal canine impaction.

Limitation of this study was that limited sample size and the convenient sampling technique could not make the results representative of the entire population. Also, this study was conducted at a single institution.

## CONCLUSIONS

In the present study, the overall prevalence of impacted canine was found lower than previous study done in similar settings. Maxillary canine impaction was more frequent than mandibular. Since the sample is not representing the whole population. Further study is recommended with large sample size from different part of the Nepal to determine overall prevalence of canine impaction.
